# Transcriptomic Plasticity in the Arthropod Generalist *Tetranychus urticae* Upon Long-Term Acclimation to Different Host Plants

**DOI:** 10.1534/g3.118.200585

**Published:** 2018-10-17

**Authors:** Simon Snoeck, Nicky Wybouw, Thomas Van Leeuwen, Wannes Dermauw

**Affiliations:** * Laboratory of Agrozoology, Department of Plants and Crops, Faculty of Bioscience Engineering, Ghent University, 9000 Ghent, Oost-Vlaanderen, Belgium; † Institute for Biodiversity and Ecosystem Dynamics (IBED), University of Amsterdam, 1012 Amsterdam, Noord-Holland, the Netherlands

**Keywords:** plant defense, host plant use, xenobiotic metabolism, single PLAT domain protein, short-chain dehydrogenase

## Abstract

The two-spotted spider mite *Tetranychus urticae* is an important pest with an exceptionally broad host plant range. This generalist rapidly acclimatizes and adapts to a new host, hereby overcoming nutritional challenges and a novel pallet of constitutive and induced plant defenses. Although recent studies reveal that a broad transcriptomic response upon host plant transfer is associated with a generalist life style in arthropod herbivores, it remains uncertain to what extent these transcriptional changes are general stress responses or host-specific. In the present study, we analyzed and compared the transcriptomic changes that occur in a single *T. urticae* population upon long-term transfer from *Phaseolus vulgaris* to a similar, but chemically defended, host (cyanogenic *Phaseolus lunatus)* and to multiple economically important crops (*Glycine max*, *Gossypium hirsutum*, *Solanum lycopersicum* and *Zea mays*). These long-term host plant transfers were associated with distinct transcriptomic responses with only a limited overlap in both specificity and directionality, suggestive of a fine-tuned transcriptional plasticity. Nonetheless, analysis at the gene family level uncovered overlapping functional processes, recruiting genes from both well-known and newly discovered detoxification families. Of note, our analyses highlighted a possible detoxification role for *Tetranychus*-specific short-chain dehydrogenases and single PLAT domain proteins, and manual genome annotation showed that both families are expanded in *T. urticae*. Our results shed new light on the molecular mechanisms underlying the remarkable adaptive potential for host plant use of generalist arthropods and set the stage for functional validation of important players in *T. urticae* detoxification of plant secondary metabolites.

Host plant acceptance by a herbivore is influenced by the nutrient composition, with the protein/carbohydrate ratio being a critical characteristic (Mattson 1980; Behmer 2009). In addition to the nutrient composition of a potential host plant, plant defenses play a pivotal role in host plant acceptance. These defenses can either be chemical or physical (*e.g.*, thorns, and trichomes). Chemical plant defense barriers include the production of toxic plant secondary metabolites and anti-digestive compounds as well as the attraction of enemies of the herbivore via the release of plant volatiles. Plants can also re-allocate their resources toward non-attacked tissues to minimize the negative fitness consequences of tissue loss due to herbivore feeding (Howe and Jander 2008; Kant *et al.* 2015).

Based on the timing of their production, plant secondary defense metabolites can be divided into two categories. Phytoanticipins are synthesized constitutively whereas phytoalexins are induced upon herbivore or pathogen attack via damage recognition and mediated by well-characterized plant hormone systems (Dixon 2001; Kant *et al.* 2015; Rioja *et al.* 2017; Stahl *et al.* 2018). Across the plant kingdom, a staggering diversity of phytoalexins and phytoanticipins have evolved in the co-evolutionary arms-race between plants and their attackers (Rosenthal and Berenbaum 1991; Wink 2010). Phytoanticipins with well-characterized effects on feeding herbivores include the glycoalkaloid tomatine in tomato, the benzoxazinoid DIMBOA-Glc in maize and related grasses and cyanogenic glucosides in cyanogenic plants, including cassava (Friedman 2002; Glauser *et al.* 2011; Dong Sub *et al.* 2014; Pičmanová *et al.* 2015). On the other hand, gossypol in cotton and isoflavonoids in legumes are examples of phytoalexins (McCormick 1982; Dakora and Phillips 1996). Another well-studied induced defense response upon herbivore feeding is the increased production of anti-digestive compounds such as proteinase inhibitors (PIs) that causes an amino acid deficiency in the attacking herbivore (Green and Ryan 1972; Hartl *et al.* 2010). Such increase of PIs has been observed in many plants upon attack of insects but also spider mite herbivores such as *Tetranychus urticae* and *Tetranychus evansi* (Kant *et al.* 2004; Martel *et al.* 2015).

Arthropod herbivores have developed several mechanisms to avoid, resist or suppress plant defenses. Two main mechanisms are thought to allow herbivores to cope with ingested plant secondary metabolites: (1) mechanisms that decrease sensitivity and (2) mechanisms that decrease exposure to plant metabolites, such as sequestration and increased metabolism (Kant *et al.* 2015). With the exception of a number of biological systems such as herbivore resistance against plant cardenolides (Dobler *et al.* 2012), the first mechanism has only been rarely documented. Probably, this is because plant secondary metabolites often have multiple or unspecific modes of action, in contrast to, for example, insecticides used to control insects and mites (for a review, see Feyereisen *et al.* 2015 and Van Leeuwen & Dermauw 2016). Mechanisms of decreased exposure, on the other hand, are far better documented and in many cases are mediated by genes that code for enzymes and transporters that typically belong to ubiquitous multi-gene families (Després *et al.* 2007; Heckel 2014; Heidel-Fischer and Vogel 2015; Erb and Robert 2016). Metabolic detoxification can be categorized into three phases based on the interaction with the ingested toxin. These interactions are: direct metabolism (phase I), conjugation (phase II) and translocation (phase III). Enzymes that operate during phase I are often cytochrome P450 monooxygenases (P450s) and carboxyl/choline esterases (CCEs), whereas enzymes such as glutathione-S-transferases (GSTs) and UDP-glycosyltransferases (UGTs) typically operate during phase II. Finally, transport of the toxins or the phase II metabolites out of the cell or into specialized cell compartments is often performed by ATP-binding cassette (ABC) transporters and solute carrier (SLC) family proteins (Brattsten 1988; Després *et al.* 2007; Dermauw and Van Leeuwen 2014; Heckel 2014; Kant *et al.* 2015). Recently it was reviewed whether the genes encoding the above-mentioned enzymes and transporters are less abundant in the genomes of specialist herbivores (those restricted to one or a few related host plants) compared to generalist herbivores (able to feed on a diverse set of host plants) and whether generalist herbivores are genetically predisposed to rapidly develop pesticide resistance; so far, conclusive evidence is yet to be found for these relationships (Rane *et al.* 2016; Dermauw *et al.* 2018; Hardy *et al.* 2018). Some studies also found that arthropod generalists exhibit a remarkably stronger transcriptional response upon host plant transfer compared to specialists and suggest that this activity is linked to the ability to cope with different host plants (Wybouw *et al.* 2015; Schweizer *et al.* 2017). More studies are however needed to establish the generality of these observations across the Arthropoda phylum.

Instead of coping with ingested plant secondary metabolites, some arthropod herbivores also evolved the ability to suppress the induced plant defenses, mostly via the secretion of molecules directly into the plant tissue (named effectors, reviewed in Kant *et al.* 2015 and Felton *et al.* 2014) and expansion of salivary genes has been suggested to be important in the adaptation processes of generalist herbivores (Jonckheere 2018; Boulain *et al.* 2018). The relative importance of plant defense manipulation and detoxification in arthropod-plant interactions remains however to be determined (Rioja *et al.* 2017; Blaazer *et al.* 2018).

The two-spotted spider mite *T. urticae* is among the most polyphagous herbivores known, with a host range covering more than 1,100 different plant species, scattered over more than 140 different plant families. Together, these plants produce a staggering number of different plant defense metabolites (Migeon, Nouguier and Dorkeld 2010; Grbić *et al.* 2011). It is well known that *T. urticae* populations readily, but differentially, adapt to a novel plant (Gould 1979; Fry 1989; Agrawal *et al.* 2002; Magalhães *et al.* 2007, 2009). Analysis of the *T. urticae* genome revealed large, lineage-specific expansions of detoxification gene families including P450s, CCEs, GSTs, UGTs and ABCs (Grbić *et al.* 2011; Dermauw *et al.* 2013a; Ahn *et al.* 2014). In addition, analysis of the spider mite salivome revealed a whole array of putative effectors (Jonckheere *et al.* 2016; Jonckheere 2018), of which two effectively suppress plant defenses and promote mite performance (Villarroel *et al.* 2016).

In recent years, many of the “classical” detoxification genes (coding for P450s, CCEs, GSTs, UGTs and ABCs) were shown to be differentially expressed upon transfer of mite populations to different host plants, but also genes previously not known to be implicated in arthropod detoxification were uncovered (Grbić *et al.* 2011; Dermauw *et al.* 2013b; Ahn *et al.* 2014; Zhurov *et al.* 2014; Wybouw *et al.* 2015). These include genes coding for binding/sequestering proteins such as lipocalins and transporters of the Major Facilitator Superfamily. Remarkably, spider mites have also acquired novel metabolic abilities via horizontal gene transfer. The horizontally transferred gene repertoire of *T. urticae* includes a family of 17 intradiol ring-cleavage dioxygenases (DOGs) capable of hydrolyzing aromatic ring structures (Dermauw *et al.* 2013b; Wybouw *et al.* 2015), but also a gene (*β-cyanoalanine synthase*) that was horizontally transferred from bacteria and of which its encoded enzyme detoxifies hydrogen cyanide (Wybouw *et al.* 2014) (see Wybouw *et al.* 2016 and Wybouw *et al.* 2018 for the general role of horizontal gene transfer in the evolution of insect and mite herbivory). The majority of these and other gene expression studies was however based on short-term transfer (less than or equal to 24 h) of plant-feeding mites to a new host (Grbić *et al.* 2011; Dermauw *et al.* 2013b; Zhurov *et al.* 2014) and only few studies have assessed mite gene expression changes upon long-term acclimation (> 1 generation) or adaptation to a new host (Dermauw *et al.*, 2013b; Wybouw *et al.*, 2014, Wybouw *et al.* 2015). Moreover, studies examining expression changes upon long-term acclimation in non-chelicerate arthropod herbivores are very scarce (Xie *et al.* 2014; Müller *et al.* 2017; Mathers *et al.* 2017). In this study, we examined the transcriptomic responses of *T. urticae* to a long-term transfer from bean to five different host plants; lima bean, soybean, cotton, tomato, and maize. We assessed the host plant specificity and overlap of these transcriptomic changes and dissected the different gene families involved, including “unexpected” families such as short-chain dehydrogenases (SDRs) and single PLAT domain proteins.

## Materials and Methods

### Plants and spider mites

The ancestral reference population (‘London’) originates from a wild-collected *T. urticae* population from the Vineland region (Ontario, Canada) and was previously described (Grbić *et al.* 2011). The London laboratory population was maintained on potted common bean plants (*Phaseolus vulgaris* L. cv. ‘Prelude’) at a continuously high population density and served as the ancestral population for all host plant transfers in the current study. Lines were established on different host plants by transferring about 250 adult females to lima bean (*Phaseolus lunatus* L. genotype 8078), soybean (*Glycine max*), maize (*Zea mays* L. cv. ‘Ronaldinio’), tomato (*Solanum lycopersicum* L. cv ‘Moneymaker’) and cotton (*Gossypium hirsutum*) (see Wybouw *et al.* 2012, 2015; Jonckheere *et al.* 2016 for a more detailed description of the experimental set-up). Three independent lines were generated on cotton and tomato, whereas four independent lines were obtained for lima bean, maize, and soybean. All lines were mass reared on their respective host plants at 26° (±0.5°), 60% relative humidity (RH) and 16/8 h light/dark photoperiod.

### RNA isolation, gene expression microarray set-up and differential gene expression analysis

Samples were collected from the soybean, cotton, and maize lines three months (approximately five generations) after transfer to the new host (Jonckheere *et al.* 2016), while the tomato and lima bean lines were collected after 18 months (approximately 30 generations) (Wybouw *et al.* 2014, 2015). Per sample, RNA was extracted from a pool of 100-120 adult females using a RNeasy minikit (Qiagen). Following DNase treatment (Turbo DN*ase*, Ambion), the concentration and integrity of the RNA samples were assessed by Nanodrop and by running 1µl on a 1% agarose gel. RNA was labeled using the Low Input Quick Amplification Kit (Agilent Technologies) following the manufacturer’s instructions. RNA that was collected from mites of the ancestral London population on common bean and of mites that were transferred to a novel host were consistently dyed with cy3 and cy5, respectively. Cyanine-labeled RNA was hybridized to a custom-made gene-expression microarray (GEO Platform GPL16890, Bryon *et al.* 2013). Hybridization, washing and scanning protocols were identical as previously described (Dermauw *et al.* 2013b). Raw intensity data were used as input for final processing and statistical analysis in limma of the Bioconductor framework (Smyth 2004). Here, background correction was first performed by the ‘normexp’ method, using an offset of 50 (Ritchie *et al.* 2007). Background-corrected data were within- and between-array normalized (global loess and Aquantile, respectively) and quality was subsequently assessed using arrayQualityMetrics (Kauffmann *et al.* 2009). Prior to final differential gene expression analysis, the 55,469 probe sequences were remapped to the *T. urticae* genome annotation of August 11, 2016 (File S1) using Bowtie2-2.2.6 with default settings (Langmead and Salzberg 2012). Only the 36,589 probes that uniquely aligned to the annotated genome were incorporated in the differential gene expression analysis. A linear model was fitted to the processed data that treated the ancestral population as a common reference (cy3 channel in sample GSM1214964-GSM1214967, GSM2124774-GSM2124784 and GSM1679383-GSM1679385). Significant differential gene-expression was identified via empirical Bayesian statistics and in reference to the ancestral population on common bean. Significant differentially expressed genes (DEGs) were identified by applying a 0.05 and 0.585 cut-off for Benjamini-Hochberg corrected p-value and absolute log_2_FC, respectively. The DEG set of each replicated host plant population was tested for enrichment of multigene families (OrthoMCL groups with at least 10 members) using a Chi square test. A Principal Component Analysis (PCA) was performed using the relative gene expression levels of all genes present on the array platform and the prcomp function within the R environment. *T. urticae* gene expression data are accessible at the Gene Expression Omnibus with accession numbers GSE50162, GSE80337 and GSE68708.

### k-means clustering

The optimal cluster number for the *k*-means clustering approach was assessed using the gap statistic (method=“global max”, seed set at 54,321, cluster number ranging from 1 to 10) (Tibshirani *et al.* 2001). The centered Pearson’s correlation was used as the distance metric for *k*-means clustering. The relative transcription levels of genes that were significantly differentially expressed in any transcriptomic comparison were used as input for *k*-means clustering. Venn-diagrams were created for both the upregulated and downregulated transcripts using the VennDiagram 1.6.20 package in the R environment.

### GO enrichment of differentially expressed genes

Gene Ontology (GO)-terms were assigned to *T. urticae* proteins using Blast2GO. The complete *T. urticae* proteome (19,086 sequences, version of August 11, 2016) was first used as query in a blastp search against the non-redundant protein database in NCBI (version of March 12, 2018) using the following settings “-outfmt 5 -evalue 1e-5 -word_size 3 -sshow_gis -num_alignments 20 -max_hsps_per_subject 20”. The resulting blastp output was then loaded into the Blast2GO (version 5.1) program and *T. urticae* proteins were annotated using the default parameters (Conesa *et al.* 2005). InterProScan 5 and ANNEX were used to augment the annotation of GO terms. GO terms were condensed using the generic GO Slim subset. Gene set enrichment analyses were conducted using the Bioconductor package piano (Väremo *et al.* 2013). The mite transcriptomic changes associated with the five host plant transfers (lima bean, soybean, cotton, tomato, and maize) were analyzed with the differential gene expression-associated statistics in a distinct directional gene set analysis (PAGE).

### OrthoMCL grouping

OrthoMCL grouping of *T. urticae* proteins was derived from Jonckheere 2018. InterProScan 5.25-64, with an E-value threshold of E^-3^, was used to identify PFAM domains in the *T. urticae* proteome (version of August 11, 2016) and PFAM domains were assigned to each OrthoMCL group based on the presence of PFAM domains in *T. urticae* proteins contained within each group. Each OrthoMCL group was filtered for those proteins of which their corresponding genes did not had probes on the microarray. For each filtered OrthoMCL protein group (having at least 5 members), we determined the percentage of corresponding genes that was differentially expressed upon long-term transfer to a host plant using the package dplyr version 0.7.4 (Wickham and Francois 2015) within the R-framework (R Developement Core Team 2015). A two-sided Fisher’s exact test in combination with the Benjamini-Hochberg procedure for multiple testing correction (FDR) using all *T. urticae* genes (having uniquely mapping probes on the array; 13,943 genes in total) as a reference was employed to identify significantly enriched OrthoMCL groups (FDR < 0.05) among the DEG sets of the different *T. urticae* host plant populations.

### Phylogenetic analysis of short-chain dehydrogenases

Among the significantly enriched OrthoMCL groups we identified two groups containing SDRs. The *T. urticae* proteome was mined for proteins with SDR-related PFAM domains; PF00106, PF01073, PF01370 and PF13561 (Persson and Kallberg 2013). Those *T. urticae* proteins with a SDR-related PFAM domain were used as query in a tblastn and blastp search (E-value threshold of E^-3^) against the *T. urticae* genome (Grbić *et al.* 2011) and proteome (version of August 11, 2018) respectively. *T. urticae* SDR gene models were modified when necessary or new SDR gene models were created using Genomeview (Abeel *et al.* 2012). *H. sapiens* SDRs were derived from (Bray *et al.* 2009), while those of *Drosophila melanogaster* and *Metaseiulus occidentalis* were identified by mining their proteomes (*M. occidentalis* 1.0 (GNOMON release, (Hoy *et al.* 2016)) and *D. melanogaster* release 6.16 (FlyBase (Gramates *et al.* 2017)), respectively) for the above-mentioned SDR-related PFAM domains (see File S2 for accession numbers). Full-length *T. urticae* SDRs were aligned with those of *M. occidentalis*, *D. melanogaster*, *Homo sapiens* and *T. urticae* using the online version of MAFFT 7 with the E-INS-i iterative refinement method strategy (Katoh *et al.* 2002), 1,000 iterations and the option “reorder”. The SDR alignment was trimmed using trimAl v1.4 with the “gappyout” option (Capella-Gutiérrez *et al.* 2009) as SDR sequences are known to be highly divergent (Persson *et al.* 2003). A phylogenetic analysis was performed on the Cipres web portal (Miller *et al.* 2010) using RAxML v8 HPC2-XSEDE (Stamatakis 2014) with the automatic protein model assignment algorithm using maximum likelihood criterion and 1,000 bootstrap replicates. The LG+G protein model was selected as the optimal model for maximum likelihood analysis. The resulting tree was midpoint rooted, visualized using MEGA6 (Tamura *et al.* 2013) and edited in CorelDRAW Home & Student ×7.

### Phylogenetic analysis of single PLAT domain proteins

OrthoMCL group Tetra_22 consisted of 20 proteins, of which three (tetur02g12320, tetur02g15207 and tetur22g02180) had a single PLAT (polycystin-1, lipoxygenase, alphatoxin) PFAM domain (PF01477) and eleven proteins belonged to the CATH/Gene3D Superfamily 2.60.60.20 (PLAT/LH2). Throughout this study, we refer to the proteins in Tetra_22 as *T. urticae* single PLAT domain proteins. *T. urticae* single PLAT domain proteins were used as query in a blastp and tblastn search (E-value threshold of E^-3^) against the *T. urticae* proteome (version of August 11, 2018) and genome (Grbić *et al.* 2011), respectively. *T. urticae* single PLAT domain protein gene models were modified when necessary or new single PLAT domain protein gene models were created using Genomeview (Abeel *et al.* 2012). The transcriptomes of related tetranychid species, *Tetranychus evansi*, *Panonychus ulmi* and *Panonychus citri* (Bajda *et al.* 2015; Villarroel *et al.* 2016) were mined for single PLAT domain protein genes using tblastn (with an E-value threshold E^-5^) and *T. urticae* single PLAT domain proteins as query. Redundant tblastn transcript hits were filtered using the cd-hit-est software (Fu *et al.* 2012) with the following settings “-c 0.95 -n 10”. Those *T. evansi*, *P. ulmi* and *P. citri* tblastn hits of more than 100 amino acids long were retained for further analysis. In addition, we also mined the NCBI non-redundant protein database (version of May 1, 2018) for the presence of these proteins in non-tetranychid species using blastp (with an E-value threshold E^-5^) and *T. urticae* single PLAT domain proteins as query (see File S3 for accession numbers). Full-length *T. urticae* single PLAT domain proteins were aligned with those of *T. evansi*, *P. ulmi* and *P. citri* using the online version of MAFFT 7 with the E-INS-i iterative refinement method strategy (Katoh *et al.* 2002), 1000 iterations and the option “reorder”. A phylogenetic analysis was performed on the Cipres web portal (Miller *et al.* 2010) using RAxML v8 HPC2-XSEDE (Stamatakis 2014) with the automatic protein model assignment algorithm using maximum likelihood criterion and 1,000 bootstrap replicates. The LG+G protein model was selected as the optimal model for maximum likelihood analysis. The resulting tree was midpoint rooted, visualized using MEGA6 (Tamura *et al.* 2013) and edited in CorelDRAW Home & Student ×7.

### Detection and analysis of short-chain dehydrogenase and single PLAT domain protein clusters

A sliding window approach earlier described in Ngoc *et al.*, 2016 was used to identify clusters of both the SDR and single PLAT domain protein genes throughout the *T. urticae* genome. A 50-kb window, incremented in 10-kb steps, was used. Only complete SDR and single PLAT domain protein genes were included in the analysis. Genes were considered as a part of each sliding window cluster if any portion of them overlapped the 50-kb window. Neighboring clusters that shared at least one gene were considered to be part of the same cluster, and were merged into a single larger cluster (as described in Thomas 2006). The midpoints of the final clusters and the number of genes within each cluster were used for plotting.

### Data availability

File S1 contains the CDS sequences of the *T. urticae* genome annotation of August 11, 2016. File S2 contains the protein sequences of the SDRs of *T. urticae*, *M. occidentalis*, *D. melanogaster* and *H. sapiens* that were included in the phylogenetic analysis. File S3 contains the sequences of the full-length single PLAT domain proteins of *T. urticae*, *T. evansi*, *P. ulmi* and *P. citri* that were included in the phylogenetic analysis. Figure S1 shows the expression heatmaps of genes coding for group I and II SDRs and of group I and II single PLAT domain protein genes across the replicated *T. urticae* host plant populations (lima bean, soybean, cotton, tomato and maize). Table S1 contains the differential gene expression results of the *T. urticae* host plant populations. Table S2 shows the overlap between DEGs of the different *T. urticae* host plant populations. Table S3 shows the *k*-means clustering of the DEGs identified in the different host plant populations of *T. urticae*. Table S4 shows the OrthoMCL grouping of the *T. urticae* proteome. Table S5 contains the OrthoMCL enrichment results of the DEG sets of each *T. urticae* host plant population. Table S6 contains the significantly enriched GO terms in the DEG sets of the different host plant populations of *T. urticae*. Table S7 contains the SDR genes annotated in the *T. urticae* genome. Table S8 contains the single PLAT domain protein genes annotated in the *T. urticae* genome. *T. urticae* gene expression data are available at the Gene Expression Omnibus with accession numbers GSE50162, GSE80337 and GSE68708. Supplemental material available at Figshare: https://doi.org/10.25387/g3.7189412.

## Results

### Effect of long-term acclimation to different host plants on the T. urticae transcriptome

**Figure 1:**
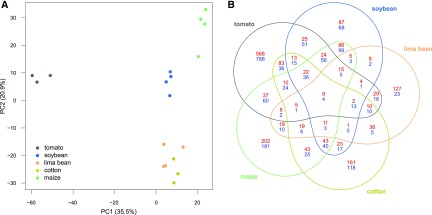
- Principal component analysis (PCA) and differential gene expression of the different host plant populations of *T. urticae*. (A) PCA plot of the relative gene expression levels in *T. urticae* populations after long-term transfer (≥ five generations) from common bean to different host plants: lima bean, soybean, cotton, tomato, and maize. (B) Venn-diagram depicting the overlap among the DEG sets of the populations after long-term transfer (≥ five generations) from common bean to different host plants. Red numbers: upregulated genes, blue numbers: downregulated genes.

Using a whole-genome gene expression microarray, we measured significant gene expression changes in *T. urticae* adult females upon long-term transfer from common bean to either lima bean, soybean, cotton, tomato or maize (log_2_FC ≥ 0.585 and Benjamini-Hochberg corrected p-value < 0.05). A PCA plot revealed that 35.5 and 20.9% of the total gene expression variation across host plant lines could be explained by PC1 and PC2, respectively (Figure 1A). Individual lines clustered by host plant on both PC1 and PC2, with PC1 clearly separating the three tomato lines from the other host plant lines. The lima bean and cotton lines clustered along PC1. Our statistical analysis showed that the host plant transfer from bean to tomato resulted in the highest number of DEGs, 1,982 DEGs in total, of which 864 were upregulated and 1,118 downregulated (Table 1). On the other hand, acclimation to lima bean resulted in the lowest amount of DEGs, 410 in total, containing 307 upregulated and 103 downregulated genes. Long-term transfer to soybean, cotton, and maize resulted in 789, 842 and 1,111 DEGs, respectively (Table 1, Table S1). In terms of amplitude, the replicated transfers to tomato and cotton plants resulted in the highest up- and downregulated DEGs. The DEG set of each replicated host plant population was enriched in multigene families (OrthoMCL groups ≥ 10 members), with 226/410, 304/789, 292/842, 479/1982 and 408/1111 of the DEGs of lima bean, soybean, cotton, tomato, and maize line belonging to multigene families, respectively (Chi-square test p-values less than E^-30^ for each DEG set). As shown in Figure 1B, the majority of DEGs was not shared between the different host plant populations, with only nine upregulated genes and four downregulated genes in common for all transfers. These common upregulated DEGs coded for an intradiol ring-cleavage dioxygenase (*tetur28g01250*), a short-chain dehydrogenase (*tetur32g01960*), two Major Facilitator Superfamily proteins (*tetur03g04330* and *tetur11g05100*), a serine protease homolog (*tetur16g03330*), a CCAAT/enhance binding protein alpha (*tetur06g04210*), a LIM-domain (PF00412) protein (*tetur06g00950*) and two hypothetical proteins (*tetur23g01600*, *tetur22g00690*). The common downregulated DEGs coded for a small secreted protein from family A (*tetur22g02750*), a viral nucleoprotein (*tetur22g01100*, which was acquired through horizontal gene transfer (Wybouw *et al.* 2018)), and two hypothetical proteins (*tetur01g09880* and *tetur13g01730*). Fifty-four genes were upregulated for four out of five transfers, while only 57 were downregulated (Table S2). Of particular note, the tomato transfer resulted in the highest number of up- and downregulated genes that were not shared with the response of any other host plant population, and therefore appeared to be the most specific response (Table 1 and Figure 1).

**Table 1:** - Differentially expressed genes in the different host plant populations of *T. urticae* (lima bean, soybean, cotton, tomato and maize) compared to an ancestral population on common bean

	total number of DEGs	upregulated DEGs	downregulated DEGs	specific upregulated DEGs* (%)	specific downregulated DEGs* (%)
lima bean	410	307	103	127 (41)	23 (22)
soybean	789	377	412	87 (23)	68 (17)
cotton	842	490	352	161 (33)	118 (34)
tomato	1,982	864	1,118	568 (66)	788 (70)
maize	1,111	557	554	202 (36)	181 (33)

* DEGs specific for a given host plant population.

### k-means clustering of transcriptomic responses to long-term host plant transfer

**Figure 2:**
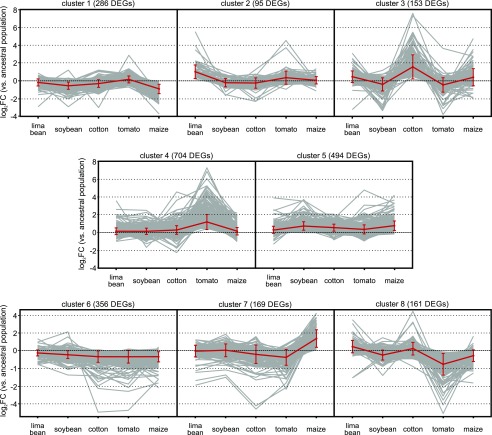
- *k*-means clustering of the *T. urticae* DEGs upon the different long-term host plant transfers. Mite transcriptomic responses to the long-term host plant transfers were categorized into eight clusters using centered Pearson’s correlation as the distance metric. Clusters were arranged according to the magnitude of log_2_FC of the DEGs. Red lines connect the averages of log_2_FC of the different host plant populations within each cluster, with error bars representing the standard deviation.

To get more insight into the global transcriptomic patterns, we performed a *k*-means clustering of the mite transcriptomic responses to the long-term host plant transfers using eight clusters (cluster number identified using the gap-statistic, Figure 2). The identity of the DEGs in each of the eight groups is listed in Table S3. Three global transcriptomic patterns became apparent when focusing on these groups. Cluster 6 and 5, with a total of 850 DEGs, appeared to reflect a general response and did not exhibit any host plant specificity. Genes of clusters 1, 3, 7, and 8, with a total of 769 DEGs, were differentially up- and downregulated upon feeding to the different hosts of this study, hereby creating zig-zag patterns. Finally, clusters 2 and 4 appeared to reflect a host plant specific transcriptional response. Cluster 2 was assembled of DEGs (n = 95) that were mainly specifically upregulated after long-term feeding on cyanogenic lima bean. This included *tetur10g01570*, a horizontally transferred gene of bacterial origin that codes for a functionally active β-cyanoalanine synthase that is able to detoxify cyanide, the main defense compound of lima bean (Wybouw *et al.* 2014). Cluster 4 consisted of the largest number of DEGs (n = 704) and largely reflected a tomato-specific transcriptional response (Figure 2, Table S3).

### Gene-set enrichment analysis

**Figure 3:**
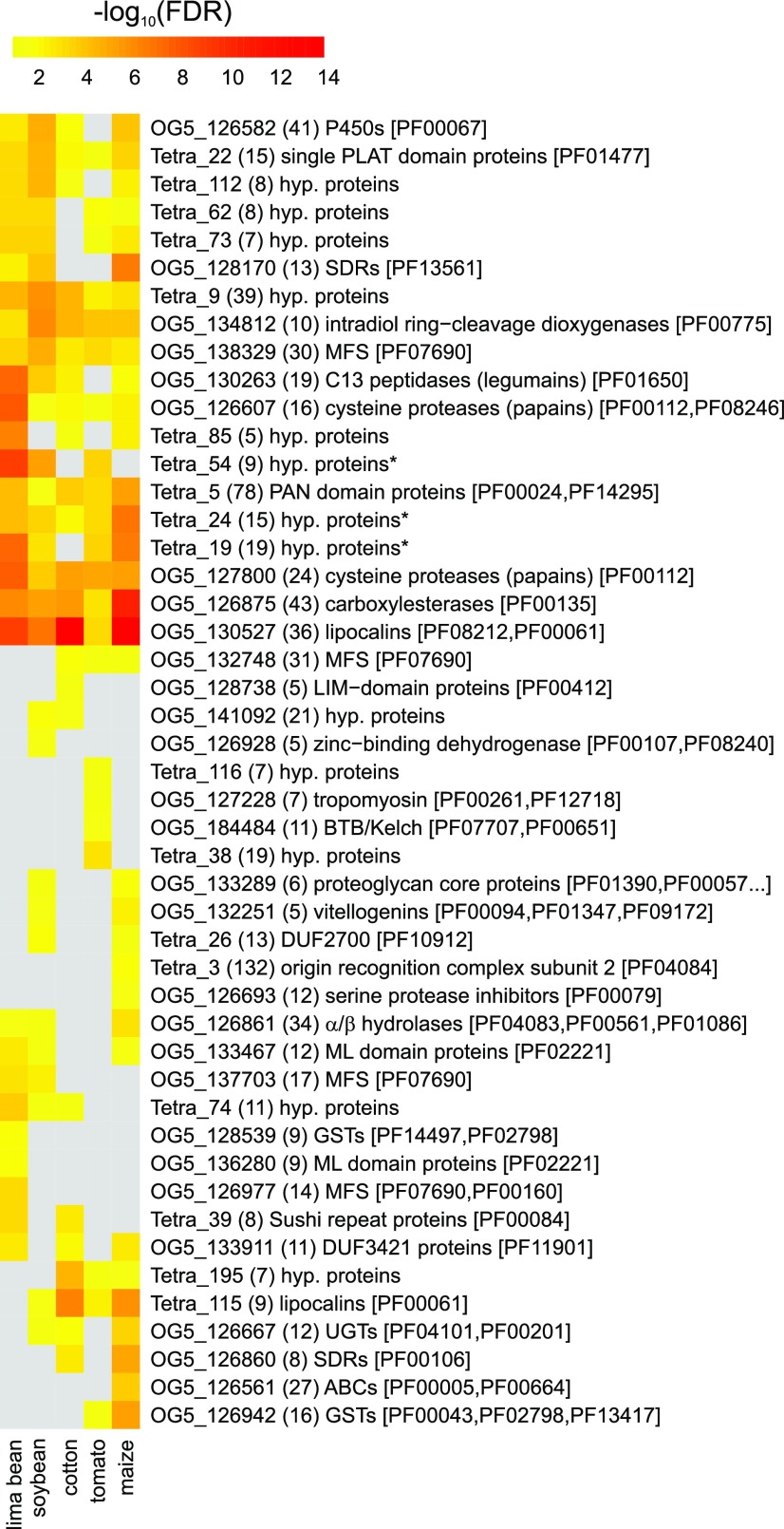
- OrthoMCL enrichment analysis of the DEGs identified in the different host plant populations of *T. urticae*. Heatmap showing the Benjamini-Hochberg corrected p-value (FDR) significance of OrthoMCL groups among DEGs of each host plant population. A gray colored cell indicates that the OrthoMCL group was not significantly enriched (FDR ≥ 0.05) for a certain host plant population. The number between parentheses represents the total number of *T. urticae* genes in an OrthoMCL group (corrected for those genes that have probes on the array), while PFAM accessions associated with any of the genes in a certain OrthoMCL group are shown between square brackets. An asterisk indicates that members of these hypothetical protein OrthoMCL groups were found in the salivary proteome of *T. urticae* (Jonckheere *et al.* 2016).

To look at gene family-wide patterns, we grouped *T. urticae* genes into OrthoMCL groups (Table S4, Figure 3), determined the percentage of DEGs for each OrthoMCL group for each replicated host plant population (Table S5) and subsequently performed an OrthoMCL enrichment analysis. Ten OrthoMCL groups were significantly enriched (FDR < 0.05) in all host plant populations: DOGs (OG5_134812), lipocalins (OG5_130527), cysteine proteases, papains (OG5_127800, OG5_126607), single PLAT domain proteins (Tetra_22), CCEs (OG5_126875), MFS proteins (OG5_138329), PAN domain proteins (Tetra_5) and hypothetical proteins (Tetra_9 (Small Secreted Protein Family A) and Tetra_24). A number of these gene groups (DOGs, lipocalins, CCEs, MFS, PAN-domain proteins, Tetra_9 (cluster 10066 in Dermauw *et al.* 2013b) and Tetra_24 (cluster 10257 in Dermauw *et al.* 2013b) were previously significantly enriched in DEG lists of both mite resistant strains and a tomato acclimatized (5 generations) mite line, while cysteine proteases and single PLAT domain proteins (cluster 10204 in Dermauw *et al.* 2013b) were only enriched in the tomato acclimatized mite line (Dermauw *et al.* 2013b). Among the remaining significantly enriched OrthoMCL groups we identified ten *T. urticae* specific gene clusters, including Tetra_19, Tetra_38, Tetra_54, Tetra_62, Tetra_73, Tetra_74, Tetra_85, Tetra_112, Tetra_116 and Tetra_195). Of particular note, members of OrthoMCL groups Tetra_19 (referred to as Tu_MCL_12 in Jonckheere *et al.* 2016), Tetra_54 (referred to as Tu_MCL_25 in Jonckheere *et al.* 2016), Tetra_62 (referred to as Tu_MCL_35 in Jonckheere *et al.* 2016, Small Secreted Protein Family F) were previously identified in the *T. urticae* salivary proteome and shown to be expressed in the salivary glands (Jonckheere *et al.* 2016). In addition, members of Tetra_54 were also shown to be constitutively upregulated in tomato-adapted mites (Wybouw *et al.* 2015). The replicated maize population had the highest number of significantly enriched OrthoMCLs (n = 33), followed by the soybean (n = 29), lima bean (n = 28), cotton (n = 24) and tomato (n = 22) populations. Four significantly enriched OrthoMCLs were unique for the tomato-fed mites, including genes coding for BTB and C-terminal Kelch related proteins (OG5_184484), while three, one, one and three significantly enriched OrthoMCL groups were unique for the lima bean, soybean, cotton, and maize populations, respectively. As a next step in our functional characterization of the mite transcriptomic responses, we complemented our OrthoMCL analysis with GO enrichment analyses (Table S6). For the DEG sets upon the lima bean, soybean, cotton, and maize long-term transfer, only a few significantly enriched GO terms could be identified, as shown in Table S6. The highest number of significantly enriched GO terms (n = 15) was found for the DEG list upon the long-term transfer to tomato, ranging from “perceiving signals” (GO:0007165) over transcription factor activity (GO:0003677 and GO:0003700) to “transmembrane transport” (GO:0055085).

### Phylogenetic analysis of *T. urticae* short-chain dehydrogenases and single PLAT domain proteins

**Figure 4:**
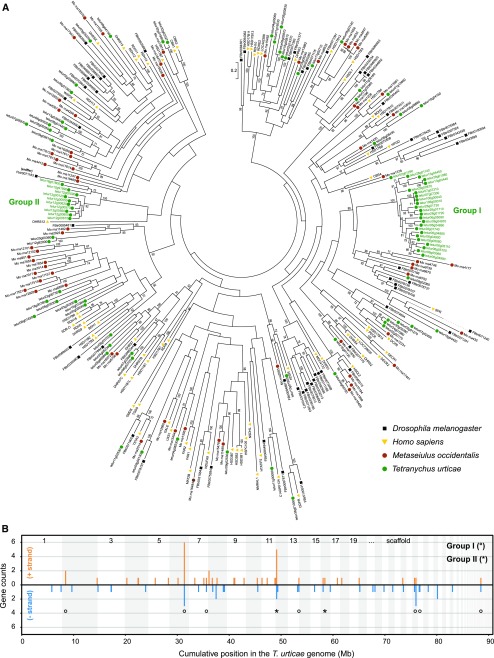
- Maximum likelihood phylogenetic analysis and genomic distribution of *T. urticae* SDRs. (A) Maximum likelihood phylogenetic analysis of the SDRs of *Homo sapiens*, *Drosophila melanogaster*, *Metaseiulus occidentalis* and *Tetranychus urticae*. Only bootstrapping values higher than 65 are shown. The scale bar represents 0.2 amino acid substitutions per site. *T. urticae* SDR expansions containing members of OrthoMCL groups that were significantly enriched among one of the DEG sets of the host plant populations are indicated by green font and labeled as Group I (°) and Group II (*). Branches that were shortened for figure clarity are shown as dashed lines. Information and accession numbers of the used SDRs can be found in Table S7 and File S2. (B) Genomic distribution of *T. urticae* SDRs is shown with lengths of vertical line segments corresponding to counts in a gene cluster; gene counts for the forward (+, orange) and reverse (−, blue) strand orientations. Clusters of SDRs were calculated such that, for a given gene, its count contributes to only one vertical line segment. Only intact SDRs were included in the analysis. Genes of the expansions of Group I and II (see panel A) are marked with their respective symbol. The genome was concatenated from largest to smallest scaffolds for display, alternating scaffolds are indicated by shading.

Among the OrthoMCL groups that were significantly enriched we identified two gene families that have not yet been associated with mite xenobiotic response to host transfer: short-chain dehydrogenases (OG5_126860 and OG5_128170, having PFAM domain PF00106 and/or PF13561) and single PLAT domain proteins (PFAM domain PF01477). Both families were annotated within the *T. urticae* Sanger-sequenced genome assembly and their phylogenetic relatedness was investigated using a maximum-likelihood phylogenetic approach. Eighty-eight full-length SDR genes and 24 SDR gene fragments/pseudogenes were annotated in the *T. urticae* genome (Table S7). Full-length *T. urticae* SDR proteins were, together with those of *M. occidentalis*, *D. melanogaster* and *H. sapiens*, used in a maximum-likelihood analysis. We identified clear 1:1:1:1 orthology between five SDRs of each arthropod species and human SDRs (HSD17B4, HSD17B8, KDSR, TSTA3, WWOX and DHRSX), verifying the validity of our phylogenetic approach. Furthermore, we identified several *T. urticae* specific expansions. Twenty-Five *T. urticae* SDRs (either belonging to OrthoMCl group OG5_128170 or OG5_136892) clustered with high bootstrap support with 5 SDRs of both *M. occidentalis* and *D. melanogaster* (group I and tetur08g02060 in Figure 4), while ten *T. urticae* SDRs (OG5_126860) clustered with high bootstrap support with a *Drosophila* SDR (FBtr0071183) (group II in Figure 4). The latter *Drosophila* SDR, named sniffer, is a carbonyl reductase and has been shown to prevent oxidative stress-induced neurodegeneration (Martin *et al.* 2011). Remarkably, OG5_128170 was significantly enriched in the DEG sets of the bean, soybean, and maize populations whereas OG5_126860 was significantly enriched in DEGs of the tomato and maize populations (Figure 3). Finally, we also identified two smaller *T. urticae* SDR expansions, one with five SDRs in *T. urticae* (belonging to OrthoMCL group OG5_127561) compared to one in *M. occidentalis* (Mo_rna15492) and one with five SDRs in *T. urticae* (belonging to OrthoMCL group OG5_131031) compared to one in both *M. occidentalis* (Mo_rna12331), *D. melanogaster* (FBtr0074654) and *H. sapiens* (DCXR). Genes encoding *T. urticae* SDRs seem to be dispersed across the genome with 61.4% of them being singletons. However, most of the genes within two *T. urticae* SDR specific expansions (Group I and II (Figure 4, panel B)) were found in clusters of scaffolds 6, 12 and 28. Within the SDR gene clusters on scaffolds 6 and 12, genes were not only found in a head-to-tail orientation but in both orientations. Moreover, the largest clusters (on scaffold 6 and scaffold 12) are rich in transposable elements (TE) sequences (see *e.g.*, *tetur12g00570* at ORCAE (http://bioinformatics.psb.ugent.be/orcae/overview/Tetur/, Sterck *et al.* 2012)), suggestive of multiple duplication events. However, features of genomic distribution will become clearer once a chromosome-wide assembly of the *T. urticae* genome will be available.

**Figure 5:**
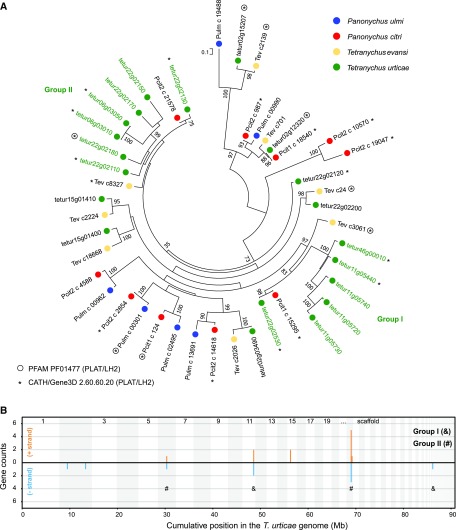
- Maximum likelihood phylogenetic analysis of tetranychid single PLAT domain proteins. (A) Maximum likelihood phylogenetic analysis of the single PLAT domain proteins of *Panonychus ulmi*, *Panonychus citri*, *Tetranychus evansi* and *Teteranychus urticae*. Only bootstrapping values higher than 65 are shown. The scale bar represents 0.1 amino acid substitutions per site. *T. urticae* single PLAT domain protein expansions are indicated by green font and labeled Group I (&) and Group II (#). Information and accession numbers of the tetranychid single PLAT domain proteins can be found in Table S8 and File S3. Those proteins with the PFAM PLAT domain (PF01477) or belonging to the CATH/Gene3D PLAT/LH2 Superfamily (2.60.60.20) are indicated with a circle and an asterisk, respectively (B) Genomic distribution of *Tetranychus urticae* single PLAT domain protein genes is shown with lengths of vertical line segments corresponding to counts in a gene cluster; gene counts for the forward (+, orange) and reverse (−, blue) strand orientations. Clusters of single PLAT domain protein genes were calculated such that a given gene its count contributes to only one vertical line segment. Only intact single PLAT domain protein genes were included in the analysis. Genes of the expansions of Group I and II (see panel A) are marked with their respective symbol. The genome was concatenated from largest to smallest scaffolds for display, alternating scaffolds are indicated by shading.

We also investigated *T. urticae* single PLAT domain proteins into more detail. Twenty-one single PLAT domain protein genes were found in the *T. urticae* genome (20 were considered as full-length genes and one as a pseudogene), and four single PLAT domain gene fragments were identified. Next, we also identified single PLAT domain protein genes in the transcriptomes of other less polyphagous tetranychid mites such as *T. evansi* (n = 8), *P. ulmi* (n = 6) and *P. citri* (n = 10) (Table S8). A blastp search against the non-redundant protein database in NCBI, revealed that tetranychid single PLAT domain proteins do not show sequence similarity with proteins of non-tetranychid eukaryotic species. A literature search, however, revealed that single PLAT domain proteins do occur in dicot and monocot plant species (Hyun *et al.* 2015), but these do not show sequence similarity with those of *T. urticae*. Nevertheless, both *T. urticae* and plant single PLAT domain proteins do share the same protein secondary structure, as they both have the PLAT domain, a β-sandwich composed of two sheets of four strands each (Bateman and Sandford 1999). Finally, we performed a maximum likelihood phylogenetic analysis using tetranychid single PLAT domain proteins (Figure 5). We identified two clear expansions of single PLAT domain proteins in *T. urticae*, with one expansion consisting of six single PLAT domain proteins in *T. urticae* (group I) compared to one in *P. citri* and *T. evansi* and one expansion consisting of seven single PLAT domain proteins in *T. urticae* (group II) compared to one in *P. citri* and *T. evansi*. Interestingly, *tetur11g05720* and *tetur11g05730* of group I showed the strongest expression changes (log_2_FC between -7 and 3) of all *T. urticae* single PLAT domain protein genes upon long-term transfer to any of the host plant lines (Table S1, Figure S1). Only 20% of the genes encoding single PLAT domain proteins in the *T. urticae* genome are singletons. The remaining single PLAT domain proteins (n = 16) are found in clusters on scaffolds 6, 11, 15 and 22 (Figure 5B). Similar as for the SDR genome distribution, single PLAT domain protein gene clusters contained genes in both orientations and were rich in TE sequences (see *e.g.*, *tetur11g05730* at the ORCAE database (Sterck *et al.* 2012)), suggestive of multiple duplication events.

## Discussion

Arthropod herbivores are important crop pests, and the last decade has seen an unprecedented increase in our understanding of the evolutionary mechanisms associated with resistance development to insecticides and acaricides used for their control. Given the wealth of knowledge on the molecular genetic mechanisms of pesticide resistance in mites and insects (Li *et al.* 2007; Feyereisen *et al.* 2015; Van Leeuwen and Dermauw 2016), it is surprising that mechanisms that allow broad plant use have remained elusive. In the last few years, an increasing number of transcriptomic studies have revealed that short-term exposure or within-generation transfer to novel hosts in polyphagous arthropod herbivores is associated with large transcriptional responses (*e.g.*, Govind *et al.* 2010; Grbić *et al.* 2011; Dermauw *et al.* 2013b; de la Paz Celorio-Mancera *et al.* 2013; Vogel *et al.* 2014; Zhurov *et al.* 2014; Roy *et al.* 2016). Fewer studies have addressed changes in gene expression upon long-term exposure and adaptation, especially in comparison to an ancestral genetic background (feeding on original host). For spider mites, Wybouw *et al.* 2015 revealed that the number of DEGs and the extent of transcriptional change increases over time and generations, and based on the functional prediction of the DEGs upon short- and long-term exposure, it was postulated that these transcriptional responses are adaptive, enabling the herbivore to survive a shift in dietary nutrients and toxins. However, the few studies addressing these important evolutionary processes looked at the transfer to a single or very few new hosts (Dermauw *et al.* 2013b; Wybouw *et al.* 2014, 2015; Xie *et al.* 2014; Müller *et al.* 2017; Mathers *et al.* 2017). Therefore, we have addressed in the current study to what extent the long-term transcriptional responses are host plant specific, using spider mites as a model. In addition, we investigated which multi-gene families were associated with the different host plant transfers.

Mites were transferred from their ancestral host (common bean) to lima bean, soybean, cotton, tomato, and maize. These plant species were selected as many of these are economically important crops on which spider mites are reported as pests (Van Leeuwen *et al.* 2015). In addition, some of the metabolites that are produced by this selection of plants have been well-characterized as plant allelochemicals with a defensive role against attacking herbivores, including the cyanogenic glucosides of lima bean, the tomato alkaloid tomatine (Friedman 2002; Dong Sub *et al.* 2014), coumestrol in soybean leaves (Yuk *et al.* 2011), the terpenoid gossypol produced by cotton (McCormick 1982) and the benzoxazinoid DIMBOA-Glc in maize (Glauser *et al.* 2011). Remarkably, although it is suggested that generalists have a less fine-tuned, host-specific regulation of gene expression compared to a specialist (Voelckel and Baldwin 2004; Govind *et al.* 2010; Dermauw *et al.* 2013b), the majority of *T. urticae* DEGs were not shared between the different host plant populations in our study. Furthermore, the number of DEGs upon the different host plant transfers could also be related to the phylogenetic distance of the novel host plant to the ancestral host (common bean, Fabaceae) (Table 1). The host specificity of the mite transcriptomic response was also reflected in our *k*-means clustering analysis, where the majority of the DEGs were present in clusters that show a host-specific pattern (Figure 2). Such transcriptomic specificity was also observed recently in the oligophagous mustard leaf beetle (*Phaedon cochleariae*) (Müller *et al.* 2017). When this beetle is transferred from its original host *Brassica rapa* to *Nasturtium officinale* and *Sinapis alba* for 26 generations, transcriptomic analysis shows that most of the response is host plant specific, even though the two new hosts share the same classes of defensive metabolites as the ancestral host (glucosinolates, phenolics, and terpenoids – in different compositions).

Wybouw *et al.* 2015 showed that approximately half of the complete transcriptional response of *T. urticae* after a long-term exposure to tomato is genetically determined and thus evolves upon tomato adaptation. The genetic changes in tomato-adapted mites affect both constitutive transcription and within-generation transcriptional plasticity. Here, we did not investigate whether the long-term transfer resulted in adaptation and therefore cannot distinguish between genetic adaptation, environmental induction and an interaction between these factors as the cause of the transcriptomic changes. Nevertheless, as the ancestral population was genetically diverse and the PCA plot did not show any signs of genetic drift, a substantial part of the observed responses probably resulted from genetic adaptation. In corroboration, *T. urticae* populations have shown great adaptive potential to a diverse set of novel hosts in addition to tomato (Gould 1979; Fry 1989; Magalhães *et al.* 2007, 2009; Wybouw *et al.* 2012).

Although the overall response was very specific on the gene level, there was much less specificity on the gene family level, which does suggest the presence of common mechanisms of acclimation and adaptation. Indeed, the set of DEGs of each host plant population was significantly enriched for genes from multigene families (OrthoMCL groups ≥ 10 members) and many of the multigene families that were significantly enriched, were previously shown to respond to xenobiotic pressure. These families were comprised of P450s and CCEs, involved in detoxification, cysteine proteases, involved in digestion, and previously unknown players in xenobiotic detoxification such as DOGs, lipocalins and MFS proteins (Dermauw *et al.* 2013b; Santamaría *et al.* 2015). The importance of the metabolic processes associated with their activities was also partially reflected in the GO enrichment analysis, where GO terms “peptidase activity”, “transferase activity” and “transmembrane transport” were enriched in the DEG sets of the host plant populations (Table S6). In addition to overall metabolic processes, these transcriptomic changes upon acclimation to different host plants also provide a first link between differential expression patterns of specific genes and known defense compounds of each host plant. Gossypol, for example, is a well-known phytoanticipin in cotton and it has been shown that UGT-glycosylation and P450-oxygenation of gossypol are important for gossypol detoxification (Mao *et al.* 2007; Krempl *et al.* 2016). Interestingly, a *CYP* and *UGT* gene were among the most highly upregulated genes when feeding on cotton (log_2_FC of 7.6 and 2.5 for *tetur07g06410* (*CYP392A1*) and *tetur04g02350* (*UGT203A2*), respectively, see Table S1). Similarly, a *UGT* (*tetur05g05020* (*UGT201B7*)) and *GST* (*tetur05g05270* (*TuGSTd15*)) were highly upregulated in the maize population (log_2_FC of 3.6 and 3.7, respectively) while downregulated or not differentially expressed in all other host plants, and might thus be involved in the detoxification of benzoxazinoids, phytochemicals that are widespread in grasses (Loayza-Muro *et al.* 2000; Wouters *et al.* 2016).

Next to the overall implication of gene families known to be involved in arthropod xenobiotic metabolism, our analyses also revealed the prominent presence of a number of gene families that have only been marginally associated with arthropod detoxification (Figure 3 and Table S5). For example, OrthoMCL analysis revealed that SDRs were significantly enriched in the DEG sets of the *T. urticae* host plant populations (Table S1). The SDR superfamily is one of the largest and most highly divergent protein superfamilies found in all domains of life (Kallberg *et al.* 2010). SDR enzymes are 250-300 amino acids long (see InterPro domain IPR020904) and are NAD(P)(H)-dependent oxidoreductases with low pairwise sequence identities. They contain at least 2 domains, a structurally conserved N-terminal region which binds NAD(H) or NADP(H) as a co-factor and a structurally variable C-terminal region that binds the substrate and contains the amino acids involved in catalysis (Bray *et al.* 2009). In contrast to the P450 superfamily, functional insights on the SDR superfamily are very scarce (Škarydová and Wsól 2012). Carbonyl-reducing enzymes (CDRs) from the SDR superfamily are known to be involved in the biosynthesis/metabolism of endogenous signaling molecules like steroid hormones and retinoids, but are as well involved in the detoxification of endobiotics and xenobiotics (Hoffmann and Maser 2007; Oppermann 2007; Škarydová and Wsól 2012). In humans, SDRs have been shown to play a central role in phase I metabolism by converting aldehydes or ketones into the corresponding alcohols, thereby reducing the overall chemical activity of their substrates (Škarydová and Wsól 2012; Ebert *et al.* 2016). In insects, the best characterized SDRs are alcohol dehydrogenases (Zhang *et al.* 2004; Mayoral *et al.* 2013; Figueroa-Teran *et al.* 2016). However, only few studies report upon the possible role of SDRs in arthropod-plant interactions. SDR genes are overexpressed in the Asian longhorn beetle *Anoplophora chinensis* upon dietary changes (Mason *et al.* 2016) and are present in the saliva of aphids, white flies and thrips (Su *et al.* 2012; Stafford-Banks *et al.* 2014). Reduction of quinone by a carbonyl reductases in the luna moth *Actias luna*, is presumably the best known example of an SDR that is involved in detoxification of a plant allelochemical (Lindroth 1991). *Actias luna* larvae are able to feed on plants of the Juglandaceae family, which contain juglone, a compound toxic to a variety of insects. Feeding larvae exhibited high carbonyl reductase and glutathione transferase activity, and these activities have been linked to the metabolism of juglone and related quinones in the plant family of the Jungladaceae (Lindroth 1989). Since SDRs have only been marginally described in both the context of host plant transfer as well as xenobiotic metabolism in mites, we have provided a survey of the SDR superfamily in *T. urticae* and identified eighty-eight full length SDRs in the genome of *T. urticae*, including several apparent species-specific expansions, which increased the diversity of the SDR repertoire. One of the expansions clustered together with a *Drosophila* SDR, named sniffer, a carbonyl reductase involved in the prevention of oxidative stress-induced neurodegeneration (Martin *et al.* 2011). The production of reactive-oxygen species is an essential part of the plant response toward herbivore attack, including those of spider mites (Santamaria *et al.* 2018). Several *T. urticae* SDRs that clustered with *Drosophila* sniffer were differentially expressed upon acclimation of *T. urticae* to different host plants (Figure 3, Figure S1, Table S1) and, hence, might play a protective role during spider mite feeding.

Next to the SDR gene family, the presence of a remarkable set of proteins containing a single PLAT domain was also evident from the OrthoMCL enrichment analysis (Figure 3). Proteins with a PLAT domain are ubiquitously present across eukaryotic species (see species distribution of PF01477 at https://pfam.xfam.org/) and PLAT domains are for example present in pancreatic triglyceride lipases (cd01755 at Conserved Domain Database (CDD)). However, short single PLAT domain proteins (less than 200 amino acids) are to our knowledge only present in plants (see EOG09360P3N at OrthoDB v9.1 and cd01754 at the Conserved Domain Database for phylogenetic distribution of these plant PLAT proteins) and apparently tetranychid mites (this study). There is virtually nothing known about the possible role of these proteins in plants. Hyun *et al.* 2015, 2014, showed that a single PLAT domain protein of *Arabidopsis* (PLAT1, AT4G39730*)* is involved in abiotic stress tolerance while in *Capsicum annuum* a single PLAT domain protein, named CaTin1, interferes with the redox balance of plants, leading to an altered response to ethylene and biotic/abiotic stress (Shin *et al.* 2004). Coker *et al.* 2005, on the other hand, showed that a single PLAT domain protein gene (*FIT-6*) is upregulated upon fire damage. In *T. urticae*, several single PLAT domain protein genes were among the DEGs with the strongest transcriptional response upon long-term host transfer, with a single PLAT gene (*tetur11g05730*) being more than 100-fold lower expressed upon long-term cotton feeding while being about 10-fold overexpressed in the maize population. Although one must be cautious when comparing genomic and transcriptomic data (*e.g.*, recent duplications and lowly expressed genes might be missed in transcriptomic data), a phylogenetic analysis using tetranychid single PLAT domain protein sequences derived from genomic (*T. urticae*) and transcriptomic data *(T. evansi*, *P. ulmi*, and *P. citri)* showed that single PLAT domain protein genes were expanded in the polyphagous *T. urticae* compared to oligophagous tetranychid species (Figure 5). Overall, it can be speculated that single PLAT domain proteins are involved in the stress response of *T. urticae* and that their expansion might have contributed to the polyphagous nature of this species.

In summary, we investigated long-term acclimation to five novel host plants in the spider mite *T. urticae*. Using different analytical tools, we uncovered that responses were specific on the individual gene level, but that similar gene families and metabolic processes were involved in host plant use. A number of surprising new gene families have entered the stage, such as genes encoding single PLAT domain proteins and short-chain dehydrogenases. Our data set identified specific enzymes that likely underlie resistance to specific plant allelochemicals and now await *in vitro* functional validation by recombinant expression in model systems like insect cells or *E. coli* and/or *in vivo* functional validation by reverse and forward genetic approaches, once they become available as robust tools for spider mite research.
